# Carbonylated Proteins as Key Regulators in the Progression of Metabolic Syndrome

**DOI:** 10.3390/antiox12040844

**Published:** 2023-03-31

**Authors:** Yuki Kitamura, Shinji Oikawa, Jie Chang, Yurie Mori, Gaku Ichihara, Sahoko Ichihara

**Affiliations:** 1 Department of Molecular and Environmental Medicine, Mie University Graduate School of Medicine, Tsu 514-8507, Japan; kitamura.yuki@jichi.ac.jp (Y.K.); s-oikawa@med.mie-u.ac.jp (S.O.); ymori@u-gifu-ms.ac.jp (Y.M.); 2 Department of Environmental and Preventive Medicine, Jichi Medical University School of Medicine, Shimotsuke 329-0498, Japan; 3 Graduate School of Regional Innovation Studies, Mie University, Tsu 514-8507, Japan; jchang@suda.edu.cn; 4 Department of Occupational and Environmental Health, Tokyo University of Sciences, Noda 278-8510, Japan; gak@rs.tus.ac.jp

**Keywords:** metabolic syndrome, adipose tissue, proteomics, oxidative stress, carbonylated proteins

## Abstract

Based on the known role of oxidative stress in the pathogenesis and progression of metabolic syndrome, we used two-dimensional gel electrophoresis with immunochemical detection of protein carbonyls (2D-Oxyblot) to characterize the carbonylated proteins induced by oxidative stress in spontaneously hypertensive rats/NDmcr-cp (CP), an animal model of metabolic syndrome. We also profiled the proteins that showed change of expression levels in their epididymal adipose tissue at the pre-symptomatic (6-week-old) and the symptomatic (25-week-old) stages of the metabolic syndrome. Two-dimensional fluorescence difference gel electrophoresis (2D-DIGE) combined with matrix-assisted laser desorption ionization time-of-flight tandem mass spectrometry (MALDI-TOF/TOF MS) was used to analyze proteins extracted from the epididymal adipose tissue. The up-regulated proteins identified at the pre-symptomatic stage were mainly associated with ATP production and redox reaction, while the down-regulated proteins found at the symptomatic stage were involved in antioxidant activity and the tricarboxylic acid (TCA) cycle. Further analysis using the 2D-Oxyblot showed significantly high carbonylation levels of gelsolin and glycerol-3-phosphate dehydrogenase [NAD^+^] at the symptomatic stage. These results suggest that reduced antioxidant capacity underlies the increased oxidative stress state in the metabolic syndrome. The identified carbonylated proteins, including gelsolin, are potential targets that may act as key regulators in the progression of the metabolic syndrome.

## 1. Introduction

The metabolic syndrome is characterized by a cluster of co-morbidities that includes abdominal obesity, hypertension, glucose intolerance and dyslipidemia [1]. The reported prevalence of the metabolic syndrome ranges from 10 to 40% worldwide and has been on the increase globally [2,3]. The etiology of the metabolic syndrome is complex and influenced by the interplay of both genetic background and environmental factors, such as smoking, a high-calorie diet, and physical inactivity [4,5]. Metabolic syndrome is a risk factor for the development of type 2 diabetes and cardiovascular diseases [6,7]. It is also a key risk factor for obesity-related cancers, regardless of the obesity status [8]. Since the metabolic syndrome is one of the world’s largest health problems, its management is a major challenge worldwide.

Obese adipose tissue shows signs of chronic inflammation based on the presence of hypertrophied adipocytes and the abundance of macrophages. Analysis of obese adipose tissues has confirmed the over-expression of NADPH oxidase and reactive oxygen species (ROS)-producing enzymes, and under-expression of antioxidant enzymes, such as catalase, glutathione peroxidase, and superoxide dismutase, with a resultant augmentation of oxidative stress state [9,10]. Furthermore, the combination of impaired ROS-producing enzymes and induction of antioxidant enzymes results in amelioration of glucose intolerance in obese mice [11]. Moreover, it has been shown that the generated ROS cause insulin resistance and abnormal adipocytokine production in adipocytes, leading to the development of the metabolic syndrome [12,13]. Indeed, the levels of various blood and urinary markers of oxidative stress have been shown to correlate positively with visceral fat area, and negatively with blood adiponectin levels in obese individuals [14,15].

The spontaneously hypertensive rat/NDmcr-cp (CP) that shows natural development of hypertension, hyperlipidemia, and non-insulin-independent diabetes mellitus has been used as an animal model of the metabolic syndrome [16]. Obese CP rats show morbid obesity compared with the lean littermates. The genetic background of CP, which carry nonsense mutation of the leptin receptor gene, was derived from a cross between the spontaneously hypertensive rats (SHR) and the obese Koletsky rats [17]. The JCR:LA–cp rat, one of the rat strains with the cp gene, develops extreme obese/insulin-resistance by 12 weeks of age [18]. The serum total cholesterol level was higher in 18 week-old CP than that of normotensive Wistar Kyoto rats (WKY), although the level was the same between 10 week-old CP and WKY [19]. Hyperlipidemia in obese CP were primarily due to hepatic hypersecretion of very-low-density lipoprotein (VLDL) without any increase in the secretion of high-density lipoprotein (HDL) [20]. Our group reported previously down-regulation of carboxylesterase 3 (CES3) associated with adipocyte lipolysis and up-regulation of monoglyceride lipase (MGLL) regulated by the peroxisome proliferator-activated receptor (PPAR) signaling pathway in the epididymal adipose tissues of obese CP [21]. In another study from our laboratories involving analysis of the liver tissues of obese CP, the involvement of the up-regulated proteins in various metabolic processes was shown, including biological regulation, catalytic activity and binding, while the down-regulated proteins were involved in endoplasmic reticulum stress-related unfolded protein response [22]. However, although oxidative stress is associated with the pathogenesis and progression of the metabolic syndrome, there is little information on the regulatory mechanism of the carbonylated proteins induced by oxidative stress in CP. Increased oxidative stress generates ROS, which participates in damaging the cytoplasmic proteins, membrane lipids and DNA. Oxidative damage to proteins causes several events; for example, it can disrupt the active sites of enzymes, affect the conformation of structural proteins, and disturb the protein function. Among the various oxidative injuries of proteins, carbonylation is the most serious modification due to its irreversible and irreparable nature [23,24].

In the present study of CP, we profiled epididymal adipose tissue proteins with altered expression levels at both the pre-symptomatic (6-week-old CP) and symptomatic (25-week-old CP) stages of the metabolic syndrome, using proteomic analysis with two-dimensional fluorescence difference gel electrophoresis (2D-DIGE). We also carried out two dimensional gel electrophoresis with immunochemical detection of protein carbonyls (2D-Oxyblot) to identify and characterize carbonylated proteins that contribute to the progression of the metabolic syndrome.

## 2. Materials and Methods

### 2.1. Experiments of Animals

CP, spontaneously hypertensive rats/lean (Lean), and WKY were purchased from the Disease Model Cooperative Research Association (Kyoto, Japan) [21] and used in this study at 6 and 25 weeks of age (*n* = 6, each). Our previous study showed that CP develop obesity, hypertension, and dyslipidemia at a young age and presented all the features of the metabolic syndrome, such as central obesity and hypertension, as well as hypertriglyceridemia and insulin resistance beyond 24 weeks of age [25]. Thus, we used CP at 6 weeks of age as a model for the pre-symptomatic/clinical stage of the metabolic syndrome and at 25 weeks of age as a model for the symptomatic/clinical stage of the metabolic syndrome. Age-matched WKY was used as a normal control group. The animals were fed a normal diet and housed under a temperature-controlled (25 °C) environment with a 12–12 h light–dark cycle. The investigation conformed to the Guide for the Care and Use of Laboratory Animals published by the US National Institutes of Health (NIH Publication No. 85–23, revised 1996) and was approved by the Committee on Laboratory Animals Utilization of Mie University (No. 22–29).

### 2.2. Measurements of Blood Pressure and Biochemical Tests

Systolic blood pressure was measured non-invasively in conscious rats by the tail-cuff method (BP-98A, MCP-1; Softron, Tokyo, Japan), as described in detail previously [26]. Briefly, rats were placed in restraining tubes and the tail-cuff probes were attached to their tails. Before and during blood pressure measurement, the rats were warmed at 36 °C in the cylindrical thermostat of the BP-98-AL machine for more than 5 min to acclimate, and then blood pressure was measured at 2-min intervals. The average of five steady-state measurements was recorded for each rat as the actual reading. The systolic blood pressure was measured the day before dissection. The body weight of each rat was weighed. Then, all rats were sacrificed at 6 and 25 weeks of age, followed by the collection of blood samples. Blood was transferred to chilled heparin-containing tubes and centrifuged. Plasma was stored at –80 °C until analysis. Plasma levels of triglyceride and glucose were outsourced to SRL (Tokyo, Japan) (*n* = 6, for 6- and 25-week-old rats).

### 2.3. Preparation of Protein Samples

Epididymal adipose tissue was obtained from 6- or 25-week-old rats, weighed, frozen immediately in liquid nitrogen, and stored at –80 °C. The tissue samples were homogenized in lysis buffer (30 mM Tris–HCl, 7 M urea, 2 M thiourea, 4% *w/v* 3-(3-cholamidopropyl) dimethylammonio)-1-propanesulfonate (CHAPS), and a cocktail of protease inhibitors, pH 8.5) and incubated for 60 min on ice. After incubation, the homogenates were centrifuged at 30,000× *g* for 30 min at 4 °C and the supernatants were collected. The protein concentration in the supernatants was determined using the Pierce 660 nm Protein Assay Kit (Thermo Fisher Scientific, Waltham, MA, USA) with bovine serum albumin as a standard.

### 2.4. 2D-DIGE Analysis

2D-DIGE using Cydye, a highly sensitive fluorescent dye, is a powerful tool to analyze differences in protein expression profiles [27]. 2D-DIGE was performed as described previously [21,22,28]. Briefly, 25 μg of total protein per sample was labeled with 200 pmol of CyDyes (GE Healthcare, Buckinghamshire, UK) and incubated on ice for 30 min in the dark. An internal standard was generated by combining equal amounts of all samples and then labeled with Cy2 dye solution. The internal standard sample with Cy2 was run on all gels, which allowed spot matching and normalization of signals from different gels [27]. Equal protein amounts of Cy2-, Cy3-, and Cy5-labeled samples were mixed and added to an equal volume of 2 × sample buffer (7 M urea, 2 M thiourea, 4% CHAPS, 130 mM dithiothreitol (DTT), 2% IPG buffer (pI 3–10; GE Healthcare), and a cocktail of protease inhibitors). After incubation on ice for 10 min in the dark, the samples were mixed with the rehydration buffer (7 M urea, 2 M thiourea, 4% CHAPS, 13 mM DTT, 1% IPG buffer, and a protease inhibitor cocktail) and applied to IPG strips (pI 3–10 NL strips, 24 cm; GE Healthcare) for rehydration for 16 h at room temperature. Isoelectric focusing was performed on the Ettan IPGphor 3 (GE Healthcare). The strips were treated by reduction and alkylation of disulfide bonds with 10 mg/mL DTT and 25 mg/mL iodoacetamide, respectively. Then, standard sodium dodecylsulfate-polyacrylamide gel electrophoresis (SDS-PAGE) was performed using 12.5% gel (Ettan DALTsix Large Format Vertical System; GE Healthcare). The next step involved scanning the gel images using the Typhoon 9400 fluorescence scanner (GE Healthcare). Intra-gel spot detection and inter-gel matching were performed using the differential in-gel analysis (DIA) and biological variation analysis (BVA) modules in DeCyder 2D software version 7.2 (GE Healthcare).

### 2.5. 2D-Oxyblot

2D-Oxyblot analysis was performed as described in detail previously [23,29]. To detect individual carbonyl-modified proteins, the epididymal adipose proteins (*n* = 4, each group) were derivatized with 2,4-dinitrophenyl hydrazine (DNPH) (Wako Pure Chemical, Osaka, Japan) and separated by two-dimensional gel electrophoresis (2DE) according to the aforementioned 2D-DIGE procedure. After 2DE, the gels were transferred onto the polyvinylidene difluoride (PVDF) membranes (Immobilon-P Transfer Membrane, Merck Millipore, Burlington, MA, USA), using the TE77 semi-dry transfer unit (GE Healthcare). The transferred membranes were blocked and incubated with anti-2,4-dinitrophenyl hydrazone (DNP) rabbit polyclonal antibody (Merck Millipore). The chemiluminescence signal was detected on X-ray films. The spot intensities of carbonylated proteins were quantified using PDQuest version 8.0 (Bio-Rad, Hercules, CA, USA). Specific oxidation was calculated by relative carbonyl level (obtained from 2D-Oxyblot) per relative protein expression (obtained from 2D-DIGE).

### 2.6. Protein Identification

For peptide mass fingerprinting, gels containing the additional load of unlabeled proteins from the epididymal adipose tissue were stained with Coomassie brilliant blue (CBB) and matched to the fluorescent 2D-DIGE images. Selected spots were picked and digested with trypsin, as described in detail previously [28,30]. The selected CBB-stained protein spots were cut out, decolorized, dehydrated, added to trypsin solution (Promega, Madison, WI, USA) and incubated overnight at 37 °C. After digestion, tryptic peptides were extracted with 45% acetonitrile/0.1% trifluoroacetic acid (TFA) and concentrated. The solutions were spotted 1:1 with saturated α-cyanohydroxycinnamic acid (Wako Pure Chemical) matrix solution, and mixed on a stainless-steel target plate. Mass analysis was performed using matrix-assisted laser desorption ionization time-of-flight tandem mass spectrometry ((MALDI-TOF/TOF MS); 4800 Plus MALDI-TOF/TOF™ Analyzer System, Sciex, Toronto, ON, Canada) with 4000 Series Explorer version 3.5 software. Protein identification was performed using the MS/MS ion search tool in ProteinPilot version 4.0 software (Sciex) with the Universal Protein Resource (UniProt; European Bioinformatics Institute, Cambridge, UK: SIM Swiss Institute Bioinformatics, Geneva, Switzerland: Protein Information Resource, Washington, DC, USA) database as the search engine.

### 2.7. UniProt Analysis

Protein ontology classification was performed by importing proteins into the protein analysis using the UniProt database (https://www.uniprot.org/, accessed on 15 February 2023) [30]. Proteins that showed changes in their expression levels at 6 and 25 weeks of age were described according to their associated molecular functions, biological processes and cellular components.

### 2.8. Statistical Analysis

Data are presented as mean ± standard error of the mean (SEM). Differences in physiological and biochemical parameters and protein spot expressions were evaluated for statistical significance by one-way analysis of variance followed by Dunnett’s post hoc test using the JMP 8.0 software (SAS Institute Inc., Cary, NC, USA). A *p* value < 0.05 was considered statistically significant.

## 3. Results

### 3.1. Changes in Body and Epididymal Adipose Tissue Weights, Blood Pressure, and Biochemical Data

**Figure 1:**
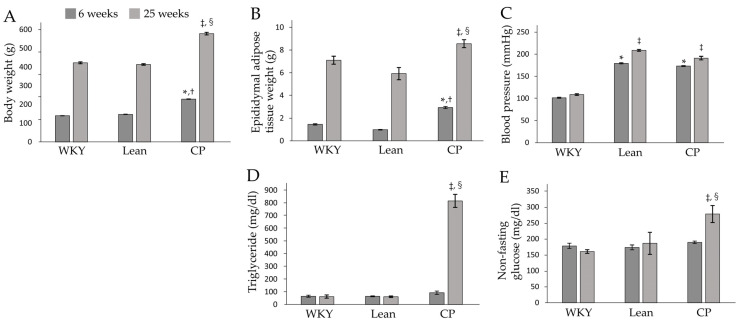
Body and epididymal adipose tissue weights, blood pressure and plasma biochemical data. (**A**) Body weight, (**B**) epididymal adipose tissue weight, (**C**) blood pressure, and plasma levels of (**D**) triglyceride and (**E**) non-fasting glucose in WKY, Lean and CP rats at 6 and 25 weeks of age. Data are mean ± SEM of six rats per group. * *p* < 0.05, compared with WKY at 6 weeks of age. ^†^ *p* < 0.05, compared with Lean at 6 weeks of age. ^‡^ *p* < 0.05, compared with WKY at 25 weeks of age. ^§^ *p* < 0.05, compared with Lean at 25 weeks of age. WKY, Wistar Kyoto rats; Lean, spontaneously hypertensive rats/lean; CP, spontaneously hypertensive rats/NDmcr-cp.

Body and epididymal adipose tissue weights were significantly higher in CP than in WKY and Lean at both 6 and 25 weeks of age (Figure 1A,B). There were no significant differences in those weights between WKY and Lean. Systolic blood pressure was significantly higher in both CP and Lean aged 6 and 25 weeks compared to WKY, and there was no significant difference in this parameter between the two strains (Figure 1C). There was no significant difference in triglyceride and non-fasting glucose levels among the three 6 weeks of age groups, while these levels were significantly higher in 25 week-old CP than in both age-matched WKY and Lean (Figure 1D,E). These results indicate that 6 weeks of age is the pre-symptomatic stage of the metabolic syndrome and that the symptomatic stage of the metabolic syndrome is established at 25 weeks of age in CP.

### 3.2. Comparison and Identification of Protein Expression

**Figure 2:**
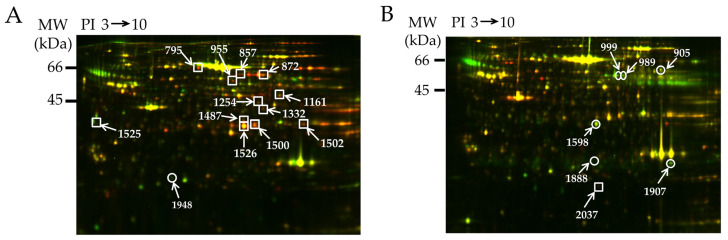
2D-DIGE gel images. Representative 2D-DIGE images of proteins in epididymal adipose tissues of (**A**) 6- and (**B**) 25-week-old WKY and CP. The proteins (25 μg) were labeled with Cy3 and Cy5 dyes, mixed, and subjected to 2D-DIGE analysis. We selected significantly different spots of 6- and 25-week-old CP compared with age-matched rats (WKY and Lean). Squares: up-regulated spots, circles: down-regulated spots.

To investigate the differentially expressed proteins in the epididymal adipose tissues of 6- and 25-week-old WKY, Lean, and CP, we performed 2D-DIGE and MALDI-TOF/TOF MS. Figure 2A shows a representative image of 2D-DIGE gel containing two samples, one labeled with Cy3 (6-week-old WKY) and the other labeled with Cy5 (6-week-old CP). Figure 2B shows a representative gel image of 25-week-old WKY and CP. The red spots on the 2D-DIGE gel indicate up-regulated proteins in CP compared with those of age-matched WKY, whereas the green spots indicate down-regulated proteins. The yellow spots indicate proteins with similar expression levels.

The 6-week-old CP rats showed 13 spots in the 2-DE gel that were significantly different compared with WKY and Lean (Figure 2A). The protein expression levels of spots 795, 857, 872, 955, 1161, 1254, 1332, 1487, 1500, 1502, 1525, and 1526 were significantly up-regulated in CP compared with WKY and Lean, while that of spot 1948 was significantly down-regulated. Based on MALDA-TOF/TOF/MS, the proteins identified in these spots were the dihydrolipoyllysine-residue acetyltransferase component of pyruvate dehydrogenase (spot 795), dihydropyrimidinase-related protein 2 (spot 857), NADP-dependent malic enzyme (spot 872), glucose-6-phosphate 1 dehydrogenase (spot 955), 6-phosphogluconate dehydrogenase decarboxylating (spot 1161), elongation factor Tu (spot 1254), long chain specific acetyl CoA dehydrogenase (spot 1332), glycerol-3-phosphate dehydrogenase [NAD^+^] (spot 1487, 1525, and 1526), aldose reductase (spot 1500), glyceraldehyde-3-phosphate dehydrogenase (spot 1502), and apolipoprotein A-1 (spot 1948) (Table 1).

The expression levels of seven protein spots were significantly different in epididymal adipose tissues of 25-week-old CP, compared with WKY and Lean (Figure 2B). The down-regulated protein spots were spots 905, 989, 999, 1598, 1888, and 1907, while the up-regulated protein spot was 2037. The identified proteins were catalase (spot 905), cytosol aminopeptidase (spot 989), d-3-phosphoglycerate dehydrogenase (spot 999), malate dehydrogenase (spot 1598), thioredoxin-dependent peroxide reductase (spot 1888), glutathione S-transferase Mu-2 (spot 1907), and ferritin light chain 1 (spot 2037) (Table 2).

### 3.3. Functional Categories of Identified Proteins

Our analysis identified 11 proteins that exhibited changes in their expression levels in 6-week-old CP (pre-symptomatic stage) and 7 proteins with changes in their expression levels in 25-week-old CP (symptomatic stage). In the next step, these proteins were imported into the UniProt database to determine their molecular functions, biological processes, and cellular components (Table 3 and Table 4). At the pre-symptomatic stage of the metabolic syndrome, the molecular functions of these proteins were mainly oxidoreductases. Annotation for the biological processes showed that the altered proteins belonged to the NADH metabolic process, the glucose metabolic process, and ATP synthesis. The cellular locations were the mitochondria and cytosol (Table 3). At the symptomatic stage of the metabolic syndrome (25-week-old CP), the molecular functions of the identified proteins were mainly antioxidants. Furthermore, annotation for the biological processes showed that the altered proteins belonged to the family of the response proteins to oxidative stress. The cellular locations were the peroxisome and the cytosol (Table 4).

### 3.4. Detection and Identification of Carbonyl Modified Proteins

**Figure 3:**
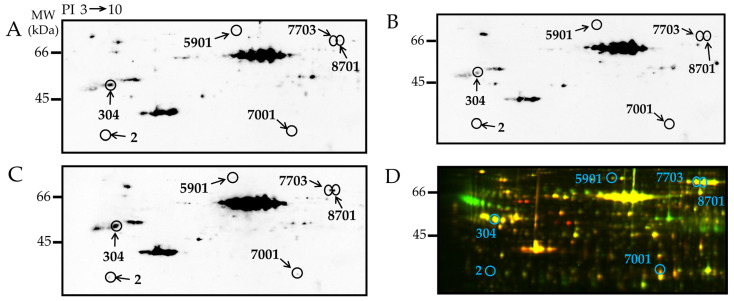
2D-Oxyblot analysis of epididymal adipose tissues of 25-week-old rats. 2D-oxyblot analysis of adipose lysates of representative (**A**) WKY, (**B**) Lean, and (**C**) CP. Proteins (100 μg) were treated with DNPH and separated by 2DE, then transferred onto a PVDF membrane. The membrane was incubated with anti-DNP primary antibody, followed by incubation with horseradish peroxidase-conjugated secondary antibody. Reaction was visualized with ECL. The obtained images were processed using PDQuest to match spots and provide spot volumes. (**D**) Blue circles: the corresponding carbonylated spots on the 2D-DIGE.

Carbonylated proteins, which were labeled with the DNP-derivates, were analyzed first by 2DE, and subsequently by Western blotting using specific antibody (2D-Oxyblot) [23,29]. Figure 3A–C show carbonylated proteins in representative images of 2D-Oxyblot from epididymal adipose tissues of the 25-week-old WKY, Lean, and CP, respectively. Among the carbonylated protein spots detected in the adipose tissues, we focused on six spots that showed significantly higher carbonylation levels in CP compared with WKY and Lean. To identify the proteins of these carbonylated spots, the 2D-Oxyblot images were matched to the corresponding CBB-stained 2DE gels, and the spots that corresponded to carbonylated proteins were resected and subjected to mass spectrometry. The corresponding spots in the 2D-DIGE image are shown in Figure 3D. The proteins identified in these spots were annexin A5 (SSP 2), tubulin beta-2B chain (SSP 304), gelsolin (SSP 5901), glycerol-3-phosphate dehydrogenase [NAD^+^] (SSP 7001), and serotransferrins (SSP 7703 and 8701) (Table 5).

**Figure 4:**
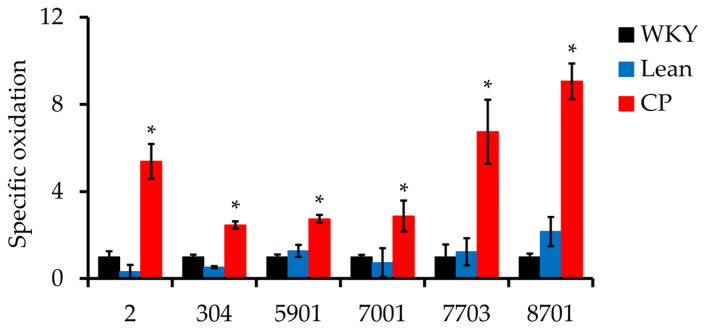
Specific oxidation of the six carbonylated protein spots of epididymal adipose tissues of 25-week-old CP. Specific oxidation was calculated from the relative carbonyl level (obtained from 2D-Oxyblot) per relative protein expression (obtained from 2D-DIGE). Data are mean relative carbonyl protein levels ± SEM of four rats per group. * *p* < 0.05 compared with WKY and Lean.

To exclude the possibility that changes in carbonylation intensity were related to changes in protein expression levels, the specific oxidation of each of the identified carbonylated protein was calculated as the relative carbonyl level (obtained from 2D-Oxyblot) per relative protein expression level (obtained from 2D-DIGE). The results showed that the expression of the six spots was significantly higher in CP compared with age-matched WKY and Lean (Figure 4).

## 4. Discussion

In the present study, we profiled adipose tissue proteins exhibiting altered expression levels between the pre-symptomatic and symptomatic stages of the metabolic syndrome. At the pre-symptomatic (6-week-old CP) stage, we identified 10 up-regulated proteins related to oxidoreductase, cytoskeleton, transferase, and the elongation factor, and one down-regulated protein related to lipid transport protein. At the symptomatic (25-week-old) stage of CP, we found five significantly down-regulated proteins, including three related to antioxidants and two related to oxidoreductases. Further analysis using the 2D-Oxyblot showed significantly increased carbonylation levels of proteins related to oxidoreductase and cytoskeleton function at the symptomatic (25-week-old) stage of CP.

Our results showed up-regulation of proteins linked to the tricarboxylic acid cycle (TCA) metabolism, electron transport chain (ETC) function, and pentose phosphate pathway at the pre-symptomatic (6-week-old) stage of CP. Among the proteins, NADP-dependent malic enzyme (ME-1), glucose-6-phosphate 1 dehydrogenase (G6PDX), and glycerol-3-phosphate dehydrogenase [NAD^+^] (GPD1) function as oxidoreductases at a molecular level and biologically in NADH or NADPH metabolic processes. The 6-phosphogluconate dehydrogenase decarboxylating (PGD) is also involved in the NADPH metabolic process and the pentose phosphate pathway. The dihydrolipoyllysine-residue acetyltransferase component of pyruvate dehydrogenase (DALT), a transferase, is known to be involved in the TCA cycle and the acetyl-CoA biosynthetic process from pyruvate. Glyceraldehyde-3-phosphate dehydrogenase (GAPDH) is involved in the carbohydrate metabolic process and microtubule cytoskeleton organization. These proteins were mainly located in the mitochondria and the cytosol. Mitochondria are important organelles known as the energy production sites in aerobic respiration, and the major cellular machinery for ROS production [31]. In the respiratory chain complexes of the mitochondrial inner membrane, ATP is produced through energy metabolism using redox reactions [32]. Our study showed that the expression of proteins related to ATP production and energy metabolism were up-regulated, probably to break down lipid at the pre-symptomatic stage of the metabolic syndrome. After that, as the symptoms progress, it is assumed that energy consumption cannot keep up with the accumulation of lipid. It is possible that this causes imbalance between lipid accumulation and lipolysis, which consequently leads to fat accumulation in the tissues. In addition, the expression of apolipoprotein A-1 (APOA1), which constitutes high-density lipoprotein (HDL), was decreased at the pre-symptomatic (6 weeks of age) stage of CP. Since HDL functions to transport lipids [33], a decrease in APOA1 may reduce the transport capacity of lipids and contribute to fat accumulation.

Fat accumulation is associated with chronic inflammation, altered glucose and lipid metabolism, adipocytokine dysregulation, and oxidative stress, which may be the early instigator of the metabolic syndrome [34]. In the pentose phosphate pathway and the TCA cycle, glucose is metabolized to produce NADH or NADPH in the process. Excess NADH or NADPH are oxidized by NADPH oxidase, which is the major regulatory enzyme in adipose tissues, to generate ROS, including superoxide and/or H_2_O_2_ in response to agonist stimulation [31,35]. Furthermore, electron transfer through the ETC interacts with molecular oxygen to form superoxide anions, as byproducts, at complex I and III [32,36]. At the pre-symptomatic stage of the metabolic syndrome, ROS might be generated due to a gradual disruption of the balance of oxidative stress. Prolonged oxidative stress directly impacts energy metabolism, including the activity of enzymes involved in the pentose phosphate pathway, the TCA cycle, and ETC [36,37], which may be responsible for the acceleration towards clinically evident metabolic syndrome.

The expression levels of proteins involved in antioxidant activity, including catalase (CAT), thioredoxin-dependent peroxide reductase (PRDX3), and glutathione S-transferase Mu-2 (GSTM2), were decreased at the symptomatic (25-week-old CP) stage of CP. Various peroxidases, such as catalase, glutathione S-transferase, and peroxiredoxins that control the levels of H_2_O_2_ in the cell, provide protection against oxidative damage by catalyzing the reduction of H_2_O_2_ into water [36,38]. NADPH is required for the reduction capacity of glutathione and thioredoxin as a proton donor [39]. Our study showed that the expression levels of oxidoreductases, including d-3-phosphoglycerate dehydrogenase (PHGDH) and malate dehydrogenase (MDH1), were also decreased at the symptomatic stage of the metabolic syndrome, which may further worsen oxidative stress. The results suggest that down-regulation of these proteins likely caused excessive oxidative stress in adipose tissues, damaging DNA, lipids, and proteins with adverse effects on cellular functions, contributing to the progression of the metabolic syndrome and its complications.

Increased oxidative stress has been reported to be involved in various pathogenic processes involved in the development of the metabolic syndrome and associated complications, including cardiovascular diseases, type 2 diabetes, obesity, hypertension, insulin resistance, and inflammation [40,41]. Indeed, oxidative stress, as assessed by serum levels of 8-hydroxy-2-deoxy-2-deoxyguanosine (8-OHdG), has been shown to be higher in patients with the metabolic syndrome than in healthy controls [42]. Oxidative damage of proteins in many tissues, consequent to acute or prolonged oxidative stress, leads to disruption of the active sites of enzymes and disturbance of protein function [23,43]. In adipose tissues, increased ROS leads to lipid hydroperoxide product and ultimately the generation of reactive lipid aldehydes, such as 4-hydroxynonenal (4-HNE) and 4-oxononenal (4-ONE) [44]. Lipid aldehydes are generally highly electrophilic, and form adducts with amino acid side chains of proteins, resulting in protein carbonylation [45,46]. In adipocytes, such modifications of proteins are associated with increased oxidative stress and metabolic dysregulation centered on mitochondrial energy metabolism. Protein carbonylation in adipocytes has been implicated in the control of insulin signaling, as well as in glucose and lipid metabolism [41,47]. In cultured adipocytes, silencing of mitochondrial glutathione S-transferase displayed increased carbonylation of several key mitochondrial proteins, including the phosphate carrier protein, NADH dehydrogenase, translocase of the inner mitochondrial membrane, and valyl-tRNA synthetase [48]. Carbonyl modification of these proteins may result in impaired mitochondrial respiration, increased superoxide production, reduced membrane potential, and decreased complex I activity [48]. Since carbonylation is the most serious modification due to its irreversible and irreparable nature among the various oxidative injuries of proteins [23,24], the present study focused on carbonylated proteins.

In the present study, proteomics analysis with 2D-Oxyblot was performed to investigate carbonylated proteins that contribute to the progression of the metabolic syndrome. The results showed that progression of obesity and development of the metabolic syndrome is associated with decreased antioxidant capacity of the adipose tissue, with a resultant increase in oxidative damage to proteins, including glycerol-3-phosphate dehydrogenase [NAD^+^] and gelsolin. Glycerol-3-phosphate dehydrogenase [NAD^+^] is an oxidoreductase involved in glycolysis [49]. Any decrease in its function due to carbonylation may induce the termination of NAD^+^ supply, resulting in abnormal glycolytic function. Gelsolin is one of the actin regulatory proteins and a member of potent actin filament proteins [50]. Low plasma levels of gelsolin have been reported in various metabolic disorders, such as cardiovascular diseases and stroke, in both animal experiments and epidemiological studies [51,52,53]. Gelsolin regulates adipocyte hypertrophy by severing actin filaments, and thus dysfunction of gelsolin through carbonylation may lead to disturbances in actin filament dynamics and hypertrophy of adipocytes. It has been reported that gelsolin knock-out mice fed a high-fat diet exhibited increased body weight and fat mass, and then presented with hepatic steatosis [54]. Furthermore, these mice showed an increase in white adipose tissue, which reflects adipocyte hypertrophy [54]. Adipocyte hypertrophy is associated with reduced secretion of leptin and adiponectin, together with enhanced secretion of insulin resistance inducers, blood coagulation promoters, monocyte chemoattractant proteins, and angiotensinogen [55,56,57]. Therefore, hypertrophy of adipocytes reflects further deterioration of the metabolic syndrome. Our findings suggest that functional changes related to carbonylated modification of glycerol-3-phosphate dehydrogenase [NAD^+^] and gelsolin by oxidative stress may contribute to the development of the metabolic syndrome. Targeting carbonylated proteins could be a potentially useful therapeutic approach in the metabolic syndrome. In recent decades, several studies have shown that various synthetic and natural substances may target carbonylated proteins/species, in order to combat the oxidative stress brought about in metabolic syndrome and its related disorders, such as diabetes, obesity, and dyslipidemia [58]. However, there is a long way from the development of technologies allowing assessment of specific carbonylated proteins as new biomarkers, and the development of new targeted therapies is even further off. Further studies are needed to identify carbonylated proteins that are potential targets of metabolic syndrome and apply them as novel therapies.

## 5. Conclusions

We have demonstrated in the present study the up-regulation of proteins related to ATP production and redox reaction at the pre-symptomatic stage of the metabolic syndrome in CP rats, as well as down-regulation of proteins with antioxidant activity at the symptomatic stage of the syndrome. Functional changes related to carbonylation of glycerol-3-phosphate dehydrogenase [NAD^+^] and gelsolin could also be involved in further worsening of the metabolic syndrome. Our results suggest that these carbonylated proteins may act as key regulators in the progression of the metabolic syndrome.

## Figures and Tables

**Table 1:** List of proteins with significant differences in their levels in epididymal adipose tissues of 6-week-old WKY, Lean, and CP.

Spot	Uniprot ID	Protein Name	% Cov.	Peptides (95%)	Fold Change Up (+) or Down (−)	
					CP/WKY	CP/Lean
795	P08461	Dihydrolipoyllysine-residue acetyltransferase component of pyruvate dehydrogenase (DALT)	19.5	7	1.39	1.54
857	P47942	Dihydropyrimidinase-related protein 2 (DRP-2)	19.2	7	1.85	1.83
872	P13697	NADP-dependent malic enzyme (ME-1)	11.9	5	1.62	1.50
955	P05370	Glucose-6-phosphate 1 dehydrogenase (G6PDX)	21.2	8	1.77	1.88
1161	P85968	6-phosphogluconate dehydrogenase decarboxylating (PGD)	19.5	6	1.58	1.67
1254	P85834	Elongation factor Tu (TUFM)	21.5	7	1.62	1.42
1332	P15650	Long chain specific acetyl CoA dehydrogenase (ACADL)	16.1	5	1.71	1.54
1487	O35077	Glycerol-3-phosphate dehydrogenase [NAD^+^] (GPD1)	35.0	9	1.42	1.61
1500	Q91W30	Aldose reductase (AKR1B8)	39.9	9	1.78	1.88
1502	P04797	Glyceraldehyde-3-phosphate dehydrogenase (GAPDH)	23.4	5	1.62	1.61
1525	O35077	Glycerol-3-phosphate dehydrogenase [NAD^+^]	42.1	9	1.52	1.73
1526	O35077	Glycerol-3-phosphate dehydrogenase [NAD^+^]	14.9	5	1.61	1.72
1948	P04639	Apolipoprotein A-1 (APOA1)	22.0	4	−1.60	−1.81

**Table 2:** List of epididymal adipose tissue proteins that showed significant changes in their expression levels in 25-week-old WKY, Lean, and CP.

Spot	Uniprot ID	Protein Name	% Cov.	Peptides (95%)	Fold Change Up (+) or Down (−)	
					CP/WKY	CP/Lean
905	P04762	Catalase (CAT)	16.9	7	−1.46	−1.39
989	Q68FS4	Cytosol aminopeptidase (LAP3)	7.9	3	−2.39	−2.58
999	O08651	D-3-phosphoglycerate dehydrogenase (PHGDH)	15.0	4	−1.69	−2.24
1598	O88989	Malate dehydrogenase (MDH1)	26.4	7	−1.66	−1.93
1888	Q9Z0V6	Thioredoxin-dependent peroxide reductase (PRDX3)	23.7	4	−1.47	−1.27
1907	P08010	Glutathione S-transferase Mu-2 (GSTM2)	36.7	7	−1.61	−2.10
2037	P02793	Ferritin light chain 1 (FTL1)	31.7	4	1.78	1.59

**Table 3:** Functional properties and location of epididymal adipose tissue proteins of 6-week-old CP that showed significant changes in their expression levels.

Protein Name	Molecular Function	Biological Process	Cellular Component
Up-regulation			
**Oxidoreductases**			
ME-1	Malate dehydrogenase (decarboxylating) (NAD^+^) activity	NADH metabolic process, NADP metabolic process, Pyruvate metabolic process	Mitochondria
G6PDX	Glucose-6-phosphate dehydrogenase activity	Glucose metabolic process, NADP biosynthetic process	Nucleus, Cytosol
GPD1	Glycerol-3-phosphate dehydrogenase [NAD(P)^+^] activity	NADH metabolic process, Glycerol-3-phosphate metabolic process, NADH oxidation, Gluconeogenesis	Cytosol
PGD	Phosphogluconate dehydrogenase (decarboxylating) activity	Carbohydrate metabolic process, NADP metabolic process	Cytosol
GAPDH	Microtubule binding, Glyceraldehyde-3-phosphate dehydrogenase (NAD^+^) activity	Carbohydrate metabolic process, Microtubule cytoskeleton organization	Nucleus, Cytosol, Cytoskeleton
ACADL	Acyl-CoA dehydrogenase activity, Fatty-acyl-CoA binding	Fatty acid catabolic process, Regulation of cholesterol metabolic process	Mitochondria
AKR1B8	Alditol: NADP^+^ 1-oxidoreductase activity	-	Mitochondria
**Transferase**			
DALT	Dihydrolipoyllysine-residue acetyltransferase activity	Tricarboxylic acid cycle, Acetyl-CoA biosynthetic process from pyruvate	Mitochondria
**Cytoskeleton**			
DRP-2	Microtubule binding, Hydrolase activity	Cytoskeleton organization	Cytosol
**Elongation factor**			
TUFM	Translation elongation factor activity	Mitochondrial translational elongation	Mitochondria
Down-regulation			
**Lipid transport**			
APOA1	Lipid transporter activity	Cholesterol metabolic process, Lipoprotein metabolic process, Reverse cholesterol transport	High-density lipoprotein particle

**Table 4:** Functional properties and location of epididymal adipose tissue proteins of 25-week-old CP that exhibited significant changes in their expression levels.

Protein Name	Molecular Function	Biological Process	Cellular Component
Down-regulation			
**Antioxidants**			
CAT	Antioxidant activity, Catalase activity	Hydrogen peroxide catabolic process, Response to hydrogen peroxide, Response to oxidative stress	Peroxisome
PRDX3	Thioredoxin peroxidase activity	Cellular response to oxidative stress, Hydrogen peroxide catabolic process, Cell redox homeostasis	Mitochondria, Cytosol
GSTM2	Glutathione peroxidase activity, Glutathione transferase activity,	Glutathione metabolic process	Cytosol, Cytoplasm
**Oxidoreductases**			
PHGDH	Phosphoglycerate dehydrogenase activity	Glutamine metabolic process, Threonine metabolic process	-
MDH1	Hydroxyphenylpyruvate reductase activity, Malate dehydrogenase activity	Tricarboxylic acid cycle, NADH metabolic process	Cytoplasm
**Hydrolase**			
LAP3	Carboxypeptidase activity, Metalloaminopeptidase activity	Proteolysis	Cytoplasm
Up-regulation			
**Iron storage**			
FTL1	Ferric iron binding	Iron ion transport, Cellular iron ion homeostasis	Cytoplasm

**Table 5:** List of proteins with increased carbonyl modification in epididymal adipose tissues of 25-week-old CP compared with WKY and Lean.

Spot	Uniprot ID	Protein Name	% Cov.	Peptides (95%)	Fold Change Up (+) or Down (−)		*p* Value
					CP/WKY	CP/Lean	
2	P14668	Annexin A5 (ANXA5)	31.4	7	5.39	16.6	0.006
304	Q3KRE8	Tubulin beta-2B chain (TUBB2B)	24.9	7	2.46	4.70	0.003
5901	Q68FP1	Gelsolin (GSN)	11.3	6	2.74	2.14	0.017
7001	O35077	Glycerol-3-phosphate dehydrogenase [NAD^+^] (GPD1)	34.1	7	2.87	3.89	0.029
7703	P12346	Serotransferrin (TF)	18.6	5	6.74	5.45	0.042
8701	P12346	Serotransferrin	19.8	7	9.06	4.19	0.005

## Data Availability

Data supporting the findings of this study are available within the paper.
